# Evaluation of the effects of *Artemisia Annua L.* and *Moringa Oleifera Lam.* on CD4 count and viral load among PLWH on ART at Mbarara Regional Referral Hospital: a double-blind randomized controlled clinical trial

**DOI:** 10.1186/s12981-024-00609-4

**Published:** 2024-04-16

**Authors:** Silvano S. Twinomujuni, Esther C Atukunda, Jackson K. Mukonzo, Musinguzi Nicholas, Felicitas Roelofsen, Patrick E. Ogwang

**Affiliations:** 1 Department of Pharmacy, Mbarara University of Science and Technology, P.O. Box 1410, Mbarara, Uganda; 2 Department of Pharmacology and Therapeutics, School of Biomedical Sciences, College of Health Sciences, Makerere University, P.O. Box 7062, Kampala, Uganda; 3 Action for Natural Medicine in the Tropics (ANAMED INTERNATIONAL), Winnenden, Germany

**Keywords:** *Artemisia annua*, *Moringa oleifera*, Immunological recovery, CD4 count, Viral load, ART

## Abstract

**Background:**

Initiation of ART among people living with HIV (PLWH) having a CD4 count ≤ 350cells/µl, produces poor immunological recovery, putting them at a high risk of opportunistic infections. To mitigate this, PLWH on ART in Uganda frequently use herbal remedies like *Artemisia annua* and *Moringa oleifera*, but their clinical benefits and potential antiretroviral (ARV) interactions remain unknown. This study examined the impact of *A. annua* and *M. oleifera* on CD4 count, viral load, and potential ARV interactions among PLWH on ART at an HIV clinic in Uganda.

**Methods:**

282 HIV-positive participants on antiretroviral therapy (ART) with a CD4 count ≤ 350cells/µl were randomized in a double-blind clinical trial to receive daily, in addition to their routine standard of care either; 1) *A. annua* leaf powder, 2) *A. annua* plus *M. oleifera*, and 3*)* routine standard of care only. Change in the CD4 count at 12 months was our primary outcome. Secondary outcomes included changes in viral load, complete blood count, and ARV plasma levels. Participants were followed up for a year and outcomes were measured at baseline, 6 and 12 months.

**Results:**

At 12 months of patient follow-up, in addition to standard of care, administration of *A. annua* + *M. oleifera* resulted in an absolute mean CD4 increment of 105.06 cells/µl, *(p* < 0.001), while administration of *A. annua* plus routine standard of care registered an absolute mean CD4 increment of 60.84 cells/µl, (*p* = 0.001) compared to the control group. The *A. annua* plus *M. oleifera* treatment significantly reduced viral load (*p* = 0.022) and increased platelet count (*p* = 0.025) and white blood cell counts (*p* = 0.003) compared to standard care alone, with no significant difference in ARV plasma levels across the groups.

**Conclusion:**

A combination of *A. annua* and *M. oleifera* leaf powders taken once a day together with the routine standard of care produced a significant increase in CD4 count, WBCs, platelets, and viral load suppression among individuals on ART. *A. annua* and *M. oleifera* have potential to offer an affordable alternative remedy for managing HIV infection, particularly in low-resource communities lacking ART access.

**Trial registration:**

ClinicalTrials.gov NCT03366922.

## Introduction

AIDS, caused by HIV, is the most prevalent immunosuppressive disease and a significant global health challenge that continues to affect and take human lives [1, 2]. Globally, an estimated 39.0 million people were living with HIV at the end of 2022, two-thirds of whom are in the WHO African Region [3]. ART effectively controls HIV replication, increasing CD4 + T counts and decreasing mortality. However, 9–40% of PLWH fail to achieve CD4 T cell count normalization, often referred to as “inadequate immunological responders” with a CD4 T cell count threshold of < 350 cells/µl at 2 years after ART initiation [4]. Despite advancements in ART access, barriers like cost, geographic location, and healthcare infrastructure persist, particularly in resource-limited countries [5] causing physicians to initiate ART when infection is advanced leading to poor immunological recovery, putting them at a high risk of opportunistic infections [6]. PLWH commonly use herbal remedies to treat HIV, and opportunistic infections, and manage the side effects of ART [7–9]. The use of herbal medicines for HIV/AIDS management is a complex issue driven by various factors such as high costs, accessibility, and perceived benefits [9]. However, more clinical research is needed to explore the clinical impact of concurrent herbal use with antiretroviral therapy, assessing patient outcomes and potential harmful interactions [10].

In Uganda, PLWH frequently use herbal medicines such as *Artemisia annua* and *Moringa oleifera* before or in addition to ART [11], and some of them have been documented to have clinical benefits [12]. *A. annua*, also known as “African Wormwood” [13], has been shown to have immunological and anti-HIV effects, and its powder has been used by some HIV patients in Uganda [14, 15]. On the other hand, *M. oleifera* is a popular nutritional supplement for HIV-infected individuals [16], with up to 80% in Africa and Uganda consuming it as a main nutritional supplement. Moringa leaves are rich in proteins, vitamins, and antioxidants, that mutually work to improve the immunity and quality of life of HIV patients [17, 18]. Despite the growing interest in *A. Annua* and *M. oleifera* for their purported immunomodulatory and antiviral properties, there is a lack of comprehensive clinical evidence regarding their effects when supplemented to ART.

This study aimed to evaluate the immunologic effects and clinical benefits of combining *A. annua* and *M. oleifera* powders with ART in PLWH, with low CD4 count (≤ 350cells/µl), providing insights into potential adjunctive treatments for optimizing HIV management.

## Methods

### Study design and setting

The study was a double-blind, randomized controlled trial conducted at Mbarara Regional Referral Hospital’s (MRRH) HIV clinic in Uganda from December 2017 to August 2020. MRRH with 350 bed capacity is located in Mbarara City, Southwestern Uganda. Every day, the hospital receives 1,200-1,500 patients including 300 HIV-positive patients from different tribal and socio-demographic locations in Uganda. The hospital was chosen as a suitable site for this study because it provides care and services to HIV- positive patients and is equipped with trained staff, nurses, counselors, physicians, pharmacists, and lab technicians.

### Recruitment of the study participants

The study involved HIV patients aged 18–66 years who had been on ART for at least one year. A Research assistant screened HIV-positive patients on ART at MRRH HIV Clinic, to identify those aged 18 years plus, with a CD4 count ≤ 350 cells/µl, living within 60 km from the clinic, having normal hematological, liver and renal function tests, and able to sign the informed consent. The study excluded pregnant women, those using other herbal or supplementary medicines, and those with pre-existing opportunistic infections. Research assistants obtained informed consent from all participants.

### Randomization, blinding, and preparation of treatments for the study participants

Participants were first stratified according to baseline CD4 levels: 350–250, 249–150, and ≤ 149 before randomization to the three groups. Using computer-generated numbers supplied by an independent biostatistician, the block randomization method was used to assign eligible participants to one of the three study groups: ART alone as the control group (CG), ART with *A. annua* group (AG), or ART with *A. annua* + *M. oleifera* group (AMG) at a 1:1:1 ratio. The study involved two herbal treatments, *A. annua* and *A. annua* + *M. oleifera*, with participants and healthcare providers blinded to the specific treatment names. This was intended to avoid bias during the study. Both intervention herbal materials underwent quality tests before use by the participants.

### Study procedures

Research assistants received opaque parcels already fixed with study codes. These parcels contained either 4 g of *A. annua* and 10 g of *M. oleifera* which were to be taken at least 8 h apart from each of the daily ARVs dosing. All participants were encouraged to take note and report all side effects.

### Preparation of study materials

Leaf powders from Ugandan plants, *A. annua* and *M. oleifera*, were authenticated by a botanist and stored in a herbarium located at Mbarara University of Science and Technology (MUST). The study pharmacist prepared and packaged *A. annua* and *M. oleifera* leaf powders in 4 g and 10 g packets, respectively, adhering to good manufacturing practices.

### Administration of treatments

Participants in the AG self-administered 4 g of *A. annua* leaf powder daily at 8 am, consumed in porridge or water, 8 h apart from their routine ARVs dosing, for 12 months. Participants in the AMG consumed 4 g of *A. annua* and 10 g of *M. oleifera* powders, mixed with porridge or water daily at 8 am, 8 h apart from their routine ARVs dosing, for 12 months. Participants in the control group received a placebo made by mixing cornstarch powder with food color, in addition to ART. Both the treatment herbs and the placebo were identically packaged and presented similarly.

Participants received one-month herbal treatments in parcels with study numbers, dosing instructions, and other safety information, including storage and safety details. Participants were reminded by an SMS to take their herbal medicine every morning between 7 and 8 am and were asked to respond with a message or call prompt. The study team collaborated with the HIV clinic to ensure that participants received regular HIV care, including ART, as prescribed. Participants were informed that herbal medicines are not replacements for ART in HIV treatment, and they were advised to continue taking prescribed ARVs as usual and correctly during the consenting process.

The study followed participants for 12 months and they were reviewed monthly by the study clinician.

### Study measurements

Blood samples were collected post-enrollment but before initiation of herbal treatments, and six and twelve months after treatment initiation to measure CD4 count, viral load, complete blood count, and liver and renal enzymes. A blood sample was taken at 1- and 2-weeks post-treatment to evaluate ARV plasma levels.

### Study outcomes

As our primary outcome, the study measured changes in absolute and relative CD4 counts after twelve months of follow-up, comparing intervention groups with control. Secondary outcomes included viral load changes, CBC, and antiretroviral plasma levels.

The study collected qualitative data on socio-demographics, herbal usage, depression, health [16], food insecurity [19], alcohol use [20], HIV stigma [21], and social support from study participants at enrolment.

### Study sample size and statistical analysis

The mean CD4 count of HIV-positive patients aged 18+, taking ART, and maintaining a mean CD4 count of ≤ 350 cells/µl for over a year at MRRH was 160 ± 110. With that mean, a sample size of 282 participants—94 in each of the three arms—was required to detect a 30% rise in CD4 counts with 80% power at 0.05 alpha, assuming a 10% loss to follow-up.

All data were cross-checked for completeness before entry into STATA Version 12 for statistical analysis, using both intention-to-treat [22] and per-protocol methods. The chi-square test statistic was used to analyze categorical data while the Wilcoxon rank-sum (Mann-Whitney) test was used to compare means between the intervention groups and the control for both the primary and secondary outcomes. The per-protocol analysis analyzed participant follow-up time and compared mean changes in CD4 count over the same follow-up time of measurements. Linear regression analysis was used to determine the impact of demographic and anthropometric parameters on the primary outcome. In all analyses, statistical significance was considered at a p-value less than 0.05. The same analysis plan was used for secondary outcomes.

### Ethical approval

The study was reviewed and approved by the MUST Research Ethics Committee (with reference number 27/05–17) and the Uganda National Council for Science and Technology (with reference number NCT03366922). The study adhered to the Helsinki Declaration’s ethical guidelines and kept all participant identities confidential, ensuring all informed consent was obtained from all participants. All treatments administered to participants have previously been documented to be safe when used for prophylaxis or as a supplement [14, 23].

### Data safety and monitoring

The data and safety monitoring committee was constituted and included senior medical scientists to ensure the safety of participating individuals. Three safety checks were done at 1 month, at 50%, and 75% of recruitment.

## Results

### Participants flow

**Fig. 1:**
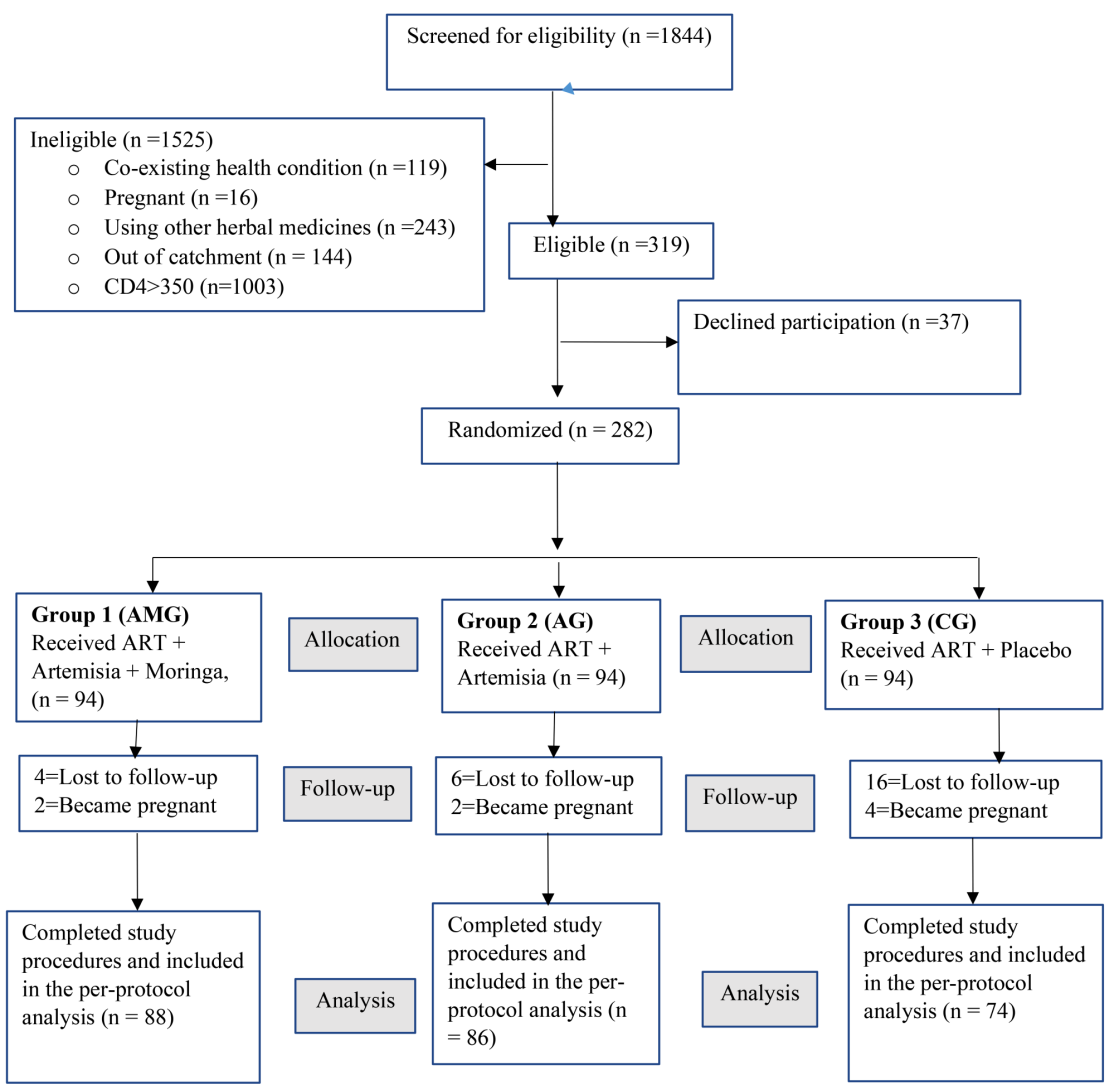
Trial flow

Out of the 1844 HIV-positive participants screened for eligibility at the MRRH HIV clinic in Southwestern Uganda, 319 were eligible. A total of 37(11.6%) declined participation in the study and 282 were randomized and enrolled to receive different treatments: The Artemisia plus Moringa group (AMG), the Artemisia group (AG), and the control group (CG). A total of 248 (87.9%) completed all study procedures, 26 (9.2%) participants were lost-to-follow-up, and 8 (2.8%) participants became pregnant and were discontinued from the study. Participants who successfully completed the study were included in the per-protocol analysis to cater for drop outs (Fig. 1).

### Demographic characteristics of the participants at enrolment

The average age was 40 years while the males dominated the three groups [CG = 49 (29.7%); AG = 56 (33.9%); AMG = 60 (36.4%)]. The majority had education above primary [CG = 68 (30%); AG = 78 (34.4%); AMG = 81(35.7%)], with irregular income, defined as consistent wages and monthly income [CG = 63 (28.1%); AG = 80 (35.7%); AMG = 81 (36.1%)]. The majority of the participants had no spouse [CG = 43 (28.3%); AG = 57 (37.5%) AMG = 52 (34.2%] with an average of 2 children per household. All the participants had similar demographic characteristics at baseline (Table 1).

**Table 1:** Baseline demographic and clinical characteristics of participants by group

Characteristic		Control Mean (SD) or n (%); (*n* = 74)	Artemisia Mean (SD) or n (%); (*n* = 88)	Artemisia + Mor. Mean (SD) or n (%) (*n* = 86)	P value
Mean Age (years)		39.2 (8.7)	40.9 (9.8)		0.32
		39.2 (8.7)		39.2 (9.9)	0.73
Gender	Male	49 (29.7)	56 (33.9)	60 (36.4)	0.69
	Female	25 (30.1)	32 (38.5)	26 (31.3)	
Education level	Primary +	68 (30)	78(34.4)	81(35.7)	0.42
	None	6 (28.6)	10 (47.6)	5 (23.8)	
Income	Regular	10 (43.5)	8 (34.8)	5 (21.7)	0.23
	Not regular	63 (28.1)	80 (35.7)	81 (36.1)	
BMI (kg/m^2^)	< 19	8 (28.6)	8 (28.6)	12 (42.9)	0.90
	19–24	51 (29.8)	61(35.7)	59 (34.5)	
	25–29	11 (27.5)	17 (42)	12 (30)	
	30+	3 (37.5)	2 (25)	3 (37.5)	
Have a spouse	Yes	12 (36.4)	12 (36.4)	9 (27.3)	0.80
	No	43 (28.3)	57 (37.5)	52 (34.2)	
	N/A	13 (31.0)	13 (31.0)	16 (38.1)	
Mean no of children/ household		2.2 (1.2)		2.6 (2.7)	0.93
		2.2 (1.2)	2.2 (2.1)		0.36
Mean duration on ART (months)		62.2 (41.1)	166.1 (1021.3		0.26
		62.2 (41.1)		53.4 (36.7)	0.19
Mean WBC (10^3^ cells/µl)		3.4 (2.7)	3.5 (2.3)		0.30
		3.4 (2.7)		4.2 (4.3)	0.23
Mean RBC (10^6^ cells/µl)		4.5 (0.8)	4.4 (0.6)		0.76
		4.5 (0.8)		4.5 (0.6)	0.33
Mean Hb level (g/dl)		14.3 (4.6)	13.7 (2.8)		0.76
		14.3 (4.6)		14.0 (2.9)	0.29
Mean CD4 count (cells/µl)		221.8 (88.9)	214.7 (86.6)		0.55
		221.8 (88.9)		227.5 (75.7)	0.89
Mean LVL		6.0 (2.1)	6.2 (2.4)		0.68
		6.0 (2.1)		6.0 (2.2)	0.63
ART regimen	AZT/3TC/EFV	20 (27.0)	24 (27.3)	23 (26.7)	0.31
	AZT/3TC/NVP	13 (17.6)	15 (17.0)	16 (18.6)	
	3TC/EFV/TDF	41 (55.4)	49 (55.6)	47 (54.7)	

ART: antiretroviral therapy; AZT: Zidovudine; BMI: body mass index; 3TC: Lamivudine; EFV: Efavirenz; LVL: log viral load; NVP: Nevirapine; RBC: red blood cells; TDF: Tenofovir, WBC: white blood cells

Data from the intervention groups and the control groups were compared using chi-square analysis for categorical variables and the Wilcoxon rank-sum (Mann-Whitney) test for continuous variables. P-values less than 0.05 were considered statistically significant

### Anthropometric and immunological characteristics of the participants at enrolment

The study participants in the three groups displayed similar baseline anthropometric and immunological characteristics. The majority of the participants had a normal BMI of 19–24 kg/m^2^ [CG = 51 (29.8%); AG = 61 (35.7%); AMG = 59 (34.5%)]. The viral load (LVL) of the majority of the patients was high [CG = 6.0 (± 2.1); AG = 6.2 (± 2.4); AMG = 6.0 (± 2.2)], with the CD4 count below < 350 cells/µl [CG = 221.8 (± 88.9); AG = 214.7 (± 86.6); AMG = 227.5(75.7)] yet they had been on ART for at least 53 months [CG = 62.2 (± 41.1); AG = 166.1 (± 1021.3); AMG = 53.4 (36.7)] before the study. The baseline haematological characteristics of the participants were the same, with efavirenz and nevirapine common to all ART regimens (Table 1).

### Effect of interventional treatments on the primary and secondary outcomes

In terms of outcomes, we noted a statistically significant difference when comparing CD4 counts at baseline with those at month 12 for each intervention group. After 12 months of patient follow-up, administration of *A. annua* + *M. oleifera* produced a significant absolute mean CD4 increment of 105.06 ± 7.25 cells/µl, *(p* < 0.001). Equally, administration of *A. annua* alone produced a lower absolute mean CD4 increment of 60.84 ± 7.20 cells/µl, (*p* = 0.001) compared to the control group (Table 2).

**Table 2:** Effect of *A. annua, and A. annua* + *M. oleifera* on CD4 count

Treatment group	Mean difference	Standard deviation	p-value	95%CI
**CD4 absolute diff at 6 months**				
CG				
AG	8.18	± 14.85	0.582	-21.07 - 37.43
AMG	12.82	± 14.97	0.860	-16.677–42.30
**CD4 absolute diff at 12 months**				
CG				
AG	60.84	± 17.20	0.001*	26.96–94.72
AMG	105.06	± 17.25	<0.001*	71.09–139.02
**CD4 relative diff at 6 months**				
CG				
AG	2.06	± 6.21	0.741	-10.18–14.29
AMG	2.70	± 6.26	0.667	-9.63–15.03
**CD4 relative diff at 12 months**				
CG				
AG	26.04	± 6.12	0.001*	13.98–38.10
AMG	30.45	± 6.14	<0.001*	18.36 − 42.54

*Statistically significant result (*p* < 0.05)

AG: Artemisia group; AMG: Artemisia plus Moringa group; CG: control group

The Wilcoxon rank-sum test was used to compare data from intervention and control groups, with p-values less than 0.05 indicating statistical significance

Furthermore, we calculated the relative difference in CD4 count. At 12 months of patient follow-up, the administration of *A. annua* + *M. oleifera* produced a relative mean CD4 increment of 30.45 ± 6.14 cells/µl (*p* < 0.001), while the administration of *A. annua* alone produced a relative mean CD4 increment of 26.04 ± 6.12 cells/µl (*p* = 0.001) compared to the control group. There was no statistically significant difference in the absolute and relative CD4 counts at 6 months in both the intervention groups compared to the control group (Table 2). Additionally, our data shows that patients who were assigned to the AMG group showed greater platelet levels (*p* = 0.025) and White Blood Cell counts (*p* = 0.003) than those who were assigned to the CG (Table 3).

**Table 3:** Effect of *A. annua, and A. annua* + *M. oleifera* on secondary outcomes after 12 months of treatment

Other secondary outcomes		Mean difference (SD) or frequency (%)	p-value
**Viral load < 50 copies, n (%)**			
CG		44 (59.5)	0.038*
AG		61(69.3)	
AMG		67 (77.9)	
**Mean LVL**			
CG		-0.75 (2.47)	
AG		-1.17 (2.32)	0.564
AMG		-2.56 (3.17)	0.022*
**Mean increment in white blood cell count**			
CG		1.21 (2.67)	
AG		1.14 (2.29)	0.080
AMG		2.08 (4.60)	0.003*
**Mean increment in platelet levels**			
CG		-7.67 (7.73)	
AG		-15.68 (10.06)	0.952
AMG		11.04 (3.29)	0.025*
**Mean plasma levels of efavirenz and nevirapine (mg/l) (SD)**			
EFV (*n* = 67)	Baseline	4.66 (0.76)	0.987
	Week 1	4.66 (0.76)	
	Week 2	4.51 (0.68)	
NVP (*n* = 38)	Baseline	7.86 (1.21)	0.991
	Week 1	7.81 (1.05)	
	Week 2	7.67 (0.94)	

* Statistically significant result (*p* < 0.05)

AG: Artemisia group; AMG: Artemisia plus Moringa group; CG: control group; EFV: efavirenz; NEV: nevirapine; VL: Viral load; LVL: log transformed viral load

Data from the intervention groups and the control groups were compared using chi-square analysis for categorical variables and the Wilcoxon rank-sum (Mann-Whitney) test for continuous variables. P-values less than 0.05 were considered statistically significant

Our findings further indicate that there was a higher proportion of participants achieving a viral load of less than 50 copies/ml in the AMG 67 (77.9%), and AG 61 (69.3%) compared to the control group (*p* = 0.038) (Table 3), while there was no significant difference in the efavirenz and nevirapine plasma levels among the participants in the intervention treatments. Whereas we did not observe a difference in the results of liver and kidney function tests and reported side effects across all the groups (results not shown), we recorded one death in the control group 5 months into the study.

### Effect of demographic, immunological, and anthropometric factors absolute CD4 count

The linear regression analysis conducted on HIV-positive patients treated with ART alone and those additionally treated with *M. oleifera* and *A. annua* revealed significant differences in absolute CD4 count. Patients in the AMG had a predicted increase of 44.22 cells/mm³ in absolute CD4 count compared to those in CG (ART only). Conversely, patients in the CG had a predicted decrease of 60.84 cells/mm³ in absolute CD4 count compared to the baseline. Furthermore, none of the demographic (age, *p* = 0.102; gender, *p* = 0.869 and income, *p* = 0.388) and anthropometric (BMI, *p* = 0.451) characteristics explored was observed to significantly influence absolute CD4 count over the study period (Table 4).

**Table 4:** Linear regression analysis of demographic, immunological, and anthropometric factors influencing absolute CD4 count

Parameter	Treatment Group	Coeff.	Std. Error	t	P value	95%CI	
						Lower limit	Upper limit
CD4 (cells/µl)	AMG	44.22	17.08	2.59	0.010*	10.52	77.93
	CG	-60.84	15.14	-4.02	0.000*	-90.74	-30.93
Age	AMG	3.39	2.06	1.64	0.102	-0.68	7.46
	CG	0.16	1.65	0.10	0.924	-3.10	3.42
Female gender	AMG	5.55	33.60	0.17	0.869	-60.79	71.89
	CG	-51.34	30.87	-1.66	0.098	-112.31	9.63
BMI (kg/m^2^)	AMG	2.81	3.72	0.76	0.451	-4.54	10.16
	CG	1.51	4.66	0.32	0.746	-7.69	10.71
Irregular income	AMG	-68.81	79.45	-0.87	0.388	-225.65	88.04
	CG	-35.02	48.48	-0.72	0.471	-130.78	60.74

*statistically significant

AG: Artemisia group; AMG: Artemisia plus Moringa group; CG: control group

Absolute CD4 cell counts of the AMG participants were compared to the CG using linear regression analysis. P-values less than 0.05 were considered statistically significant

## Discussion

After 12 months of patient follow-up, we observed significant differences in CD4 counts between the intervention and control groups. When compared to the AG and the CG, the AMG exhibited a significant absolute mean CD4 increment. Conversely, while the AG’s absolute mean CD4 increase was smaller than that of the AMG, it was still significantly higher than that of the CG (Table 2). The difference in CD4 counts across the intervention groups implies that, in comparison to *A. annua* alone or conventional care, the combined use of *A. annua* and *M. oleifera* may have had a synergistic impact in increasing immunological response. This outcome may arise from the possible changes in the biological effects and/or bioavailability of individual phytochemicals upon combination. Many combinations of pure bioactive compounds or plant extracts, each rich in phytochemicals, have been shown in numerous studies to exhibit synergistic effects [24, 25]. These combinations can result in enhanced biological activities, such as improved antioxidant properties, anti-inflammatory effects, and increased efficacy in combating various health conditions [26, 27]. This is a very useful finding because these two plants can be used as adjuvants to ART to improve health outcomes among PLWH since the plants can be cultivated in people’s homes. Both these plants have previously been documented to be safe when used for prophylaxis or as a supplement [23], and so this combination may provide a readily available and affordable source of supplement that can improve suppression of HIV replication and enabling CD4 regain to restore the body’s ability to fight against opportunistic infections [5].

Although the interventions increased CD4 count, this increase was gradual and only reached statistical significance in month 12, implying that the impact of the interventions on immune reconstitution takes time to manifest, which offers insights into the immune response in the individuals receiving *A. annua* and *M. oleifera* alongside ART. This discovery is especially significant because CD4, whose depletion is a hallmark of HIV infection, is a critical indicator of the health and efficiency of the immune system. The results also imply that the combined intervention may increase immunity over the long run, because plant-based medicine has the characteristics of slow onset [28], potentially providing a more all-encompassing strategy for HIV management by actively promoting immunological responses in addition to inhibiting viral replication with ART.

It should also be noted that, as one of the inclusion criteria, this study investigated HIV-positive individuals who had been on ART for at least a year but had a CD4 count below 350 cells/µl. Studies show that individuals who initiate ART with a CD4 count < 350 cells/µl often struggle to achieve CD4 counts > 500 cells/µl after up to 10 years on ART [29], and this might explain the slow immunological recovery.

HIV clinicians have long relied on CD4 as a disease progression predictor [30], but recent guidelines recommend viral load testing as the preferred monitoring strategy for clinical response to ART [31]. After a year of follow-up, participants in the AG and the AMG showed a statistically significant reduction in their viral load when compared to those receiving standard care. This is a promising and clinically relevant finding in the context of managing HIV infection. Reducing viral load is a major treatment objective in the management of HIV/AIDS since it is a crucial indicator of the quantity of HIV in the blood. Given that the viral load of both intervention groups significantly decreased, it is possible that using *M. oleifera*—alone or in conjunction with *A. annua* —has antiviral properties. This finding can be collaborated by a study done by [32], who concluded that leaf infusion of *M. oleifera* has, among other things, antivirus properties. In addition, *M. oleifera* is rich in bioactive substances that have been shown to have positive effects on health [33, 34]. As such, *A. annua* may be complementing it to potentially enhance the immune response [35] and contribute to viral suppression.

We further observed that treating HIV patients with *A. annua* and *M. oleifera* alongside ART did not affect their efavirenz and nevirapine plasma levels. This is an important finding regarding the safety and effectiveness of concurrent herbal medicine use in HIV-positive patients who receive these antiretroviral medications for the management of HIV. For these medications to be effective in inhibiting viral replication and halting the emergence of drug-resistant viruses, their plasma levels must remain unchanged. This lack of interaction is worth noting because it implies that the co-administration of *A. annua* and *M. oleifera* does not disrupt the pharmacokinetics of these antiretroviral drugs and hence affects their efficacy. These findings are supported by observations from previous studies that demonstrated no statistically or clinically significant interaction following 14 days of nevirapine and Moringa co-administration [16].

We also investigated the effect of demographic and anthropometric factors on the primary outcome (absolute CD4 counts) over the treatment period in the CG and the AMG (Table 4). The study found no evidence of a significant relationship between the absolute CD4 count, age, income, BMI, or gender. This suggests that the CD4 levels in this patient population may not be directly impacted by these factors. At enrollment, the majority of the participants (69%) were within the normal BMI range of (19–24 kg/m^2^) while only 3% had a BMI > 30 kg/m^2^. Our results agree with a study carried out by Cianflone et al. in which they concluded that BMI > 30 kg/m^2^ was not beneficial in the ART era and was associated with smaller CD4 count increases [36]. Additionally, our study is in agreement with that of Bahemana et al. who found no difference in long-term CD4 recovery by age at ART initiation [37].

Finally, as a secondary outcome, participants in the AMG achieved a statically significant increase in the platelet count compared to the those in the AM and CG. Changes in platelet count might have therapeutic implications, particularly in patients with HIV infection who may be at risk of thrombocytopenia. Platelets are essential for immunological response and hemostasis.

Our study had several strengths. The study successfully maintained blinding to treatment allocation, despite the bitterness of *A. Annua*, by combining porridge with the interventions during dozing. The study utilized dried plant powders, prepared by an independent pharmacist, and consumed at least 8 h apart from routine ART dosing to prevent interaction. We studied *A. annua* and *M. oleifera* which offer an affordable alternative remedy for managing HIV infection, particularly in low-resource communities lacking ART access.

Despite the strengths pointed out, our study had some important limitations. The control arm experienced a higher dropout rate due to challenges like transportation and food insecurity, which were not adequately addressed through SMS reminders. Further, we did not maintain objective measures of ART adherence or evaluate food intakes using methods like dietary records or 24-hour recall. The ARV interaction study had limitations, including a short period for potential interactions with herbs and food supplements, potentially reducing concentrations.

Finally, we recruited relatively healthy PLWH, and therefore our results may not be generalizable for individuals with renal, and liver function problems, existing opportunistic infections, or pregnant.

## Conclusion and recommendation

The study found that daily consumption of *M. oleifera* and *A. annua* leaf powders improves CD4 count, viral load suppression, and platelet count among PLWH on ART, without affecting efavirenz and nevirapine plasma levels. The findings from this study provides valuable insights into the potential benefits of *M. oleifera* and *A. annua* supplementation in HIV-positive patients on ART as adjuvant therapy. We recommend further research to rigorously measure and evaluate ART adherence by the participants. Further research is needed to determine the long-term impact of *M. oleifera*-*A. annua* combination on CD4 count, viral load, liver and kidney enzymes, and ARV plasma levels. The authors also recommend further studies of these interventions on wound healing and reduction of opportunistic infections among PLWH on ART.

## Data Availability

The datasets used and/or analyzed during this study are available from the corresponding author upon reasonable request.

## Abbreviations

AIDS: Acquired immune deficiency syndrome
AMAMED: Action for Natural Medicine in the Tropics
AMG: Artemisia + Moringa group
ART: Antiretroviral therapy
AG: Artemisia group
BMI: Body mass index
CD4: Cluster de differentiation 4
CG: Control group
CI: Confidence interval
HIV: Human immunodeficiency virus
MRRH: Mbarara Regional Referral Hospital
MUST: Mbarara university of science and technology
PLWH: People living with HIV
SD: Standard deviation
